# Pressure passivity of cerebral mitochondrial metabolism is associated with poor outcome following perinatal hypoxic ischemic brain injury

**DOI:** 10.1177/0271678X17733639

**Published:** 2017-09-26

**Authors:** Subhabrata Mitra, Gemma Bale, David Highton, Roxanna Gunny, Cristina Uria-Avellanal, Alan Bainbridge, Magdalena Sokolska, David Price, Angela Huertas-Ceballos, Giles S Kendall, Judith Meek, Ilias Tachtsidis, Nicola J Robertson

**Affiliations:** 1 Institute for Women’s Health, University College London, London, UK; 2 Department of Medical Physics and Biomedical Engineering, University College London, London, UK; 3 Neurocritical Care, National Hospital for Neurology & Neurosurgery, University College London, London, UK; 4 Paediatric Neuroradiology, Great Ormond Street Hospital for Children, London, UK; 5 Department of Medical Physics and Biomedical Engineering, University College London Hospital, London, UK; 6 Neonatal Unit, University College London Hospital, London, UK

**Keywords:** Perinatal hypoxia, near infrared spectroscopy, metabolism, cerebral hemodynamics, cerebral autoregulation

## Abstract

Hypoxic ischemic encephalopathy (HIE) leads to significant morbidity and mortality. Impaired autoregulation after hypoxia-ischaemia has been suggested to contribute further to injury. Thalamic lactate/N-Acetylasperate (Lac/NAA) peak area ratio of > 0.3 on proton (^1^H) magnetic resonance spectroscopy (MRS) is associated with poor neurodevelopment outcome following HIE. Cytochrome-c-oxidase (CCO) plays a central role in mitochondrial oxidative metabolism and ATP synthesis. Using a novel broadband NIRS system, we investigated the impact of pressure passivity of cerebral metabolism (CCO), oxygenation (haemoglobin difference (HbD)) and cerebral blood volume (total haemoglobin (HbT)) in 23 term infants following HIE during therapeutic hypothermia (HT). Sixty-minute epochs of data from each infant were studied using wavelet analysis at a mean age of 48 h. Wavelet semblance (a measure of phase difference) was calculated to compare reactivity between mean arterial blood pressure (MABP) with oxCCO, HbD and HbT. OxCCO-MABP semblance correlated with thalamic Lac/NAA (*r* = 0.48, *p* = 0.02). OxCCO-MABP semblance also differed between groups of infants with mild to moderate and severe injury measured using brain MRI score (*p* = 0.04), thalamic Lac/NAA (*p* = 0.04) and neurodevelopmental outcome at one year (*p* = 0.04). Pressure passive changes in cerebral metabolism were associated with injury severity indicated by thalamic Lac/NAA, MRI scores and neurodevelopmental assessment at one year of age.

## Introduction

Intrapartum hypoxic-ischemic injury leading to hypoxic ischaemic encephalopathy (HIE) is a significant cause of neonatal morbidity and mortality. Each year across the world, approximately 1 million babies die following intrapartum complications.^1^ Although therapeutic hypothermia improves neurodevelopmental outcome in HIE,^2^ 40–79% of cooled infants die or develop significant disability in the developed world.^3–5^ The evolution of injury following hypoxia-ischaemia has been studied using magnetic resonance spectroscopy (MRS); despite the initial recovery of cerebral energetics after resuscitation, there is a decline in energy state with increased thalamic lactate and reduced N acetyl aspartate (NAA) over the hours and days following birth. These metabolic changes have been termed secondary energy failure. These MRS studies were important for the concept that interventions such as cooling ameliorate the subsequent secondary energy failure.^6–9^ Currently, thalamic lactate/NAA peak area ratio acquired between day 5 and 14 predicts outcome in HIE using a cut off threshold of 0.3;^10^ this ratio is used with conventional MRI for counselling and prognosis.^11^

There has been recent interest in the use of blood biomarkers^12^ and monitoring cerebral autoregulation following HIE,^13–17^ but direct assessment of cerebral mitochondrial function in relation to cerebrovascular reactivity has not been investigated so far.

Cytochrome-c-oxidase (CCO) is the terminal electron acceptor inside the mitochondrial electron transport chain (ETC). It plays a crucial role in mitochondrial oxidative metabolism and is responsible for more than 95% of ATP synthesis.^18^ Using a broadband NIRS system, concentration changes in the oxidation state of CCO (oxCCO) can be measured along with changes in oxy- and deoxy-haemoglobin (HbO_2_ and Hb), with derived changes in haemoglobin difference (HbD=HbO_2_−Hb) and total haemoglobin ((HbT=HbO_2_+Hb).^19^ Changes in [oxCCO] indicate the status of the mitochondrial function and has been used to monitor the cerebral energy state following HIE in preclinical^20,21^ and clinical studies.^22–24^

Cerebral autoregulation (CA) maintains a constant cerebral blood flow (CBF) over a range of cerebral perfusion pressure and protects the brain from hypo- and hyperperfusion. Cerebrovascular circulatory function is controlled through neural, myogenic and metabolic mechanisms. Cerebral vasoparalysis leading to abnormal cerebral haemodynamics and impaired CA following HIE was associated with poor outcome in pre-hypothermic era.^25–27^ In recent years, further attempts have been made to examine the cerebrovascular reactivity status of the brain and its relationship with outcome in neonates with HIE who underwent therapeutic hypothermia, using NIRS-based haemodynamic indices.^13–16^ A close relationship between dis-autoregulation and abnormal cerebral metabolism has been described in adults after traumatic brain injury but pressure passivity was not related to CBF.^28^

Disturbances in cerebral oxidative metabolism following HIE are well documented.^7–8^ Both preclinical and clinical studies using phosphorus magnetic resonance spectroscopy (^31^P MRS) have demonstrated the depletion in cerebral energy state immediately after the HI insult (primary energy failure) followed by a further phase of deterioration 6–24 h after HI (secondary energy failure). During this secondary phase, phosphocreatinine (PCr) and neucleotide triphosphate (NTP) fell and inorganic phosphate increased (Pi) despite maintenance of adequate oxygenation and circulation. The secondary phase marked by the onset of seizures, cytotoxic oedema, accumulation of cytokines and mitochondrial failure that leads to further call death.^29^ The degree of energy failure influences the type of cell death.^30,31^ These findings further raise the importance of reviewing the relationship of a metabolic reactivity index with outcome following HIE. A cot side metabolic reactivity index using CCO has never been examined before. This is particularly intriguing in the current era, in view of the influence of HT on other early prognostic biomarkers. The predictive ability of amplitude integrated EEG^32^ and the neurological examination at 72 h are influenced by hypothermia.^33^ Thoresen and coworkers^34,35^ also demonstrated that the cerebral resistance index (RI) on Doppler ultrasound has lost the predictive value during HT. In the pre-HT era, resistance index (RI) < 0.55 was found to be a predictor of adverse outcome at 18 months following HIE in 84% of normothermic infants while it predicted outcome in only 60% on infants on day 2 during HT.^35^

Several methodologies have been used to assess cerebrovascular reactivity in both term and preterm infants.^36,37^ Both transcranial Doppler and NIRS-derived reactivity indices examined the relationship between spontaneous slow wave oscillations (0.003–0.05 Hz) in mean arterial blood pressure (MABP), Doppler flow velocity,^37^ cerebral blood volume (CBV)^16^ and cerebral oxygenation^38–42^ in the time domain. Similarly, coherence and gain have been used in frequency domain analysis.^43^ One of the major limitations for these techniques is the assumption of stationary relationship between the variables. Cerebrovascular autoregulation is dynamic, nonstationary and the signals vary both in time and frequency, more under pathological conditions.^44^ Wavelet-based analysis can overcome this issue and characterise autoregulation with improved time–frequency resolution following brain injury both in adults^45,46^ and the newborn.^17^

We hypothesised that a metabolic reactivity index based on wavelet analysis of slow wave (SW) oscillations of oxCCO and MABP measured at 48 h after birth – (a) will correlate with thalamic lactate/NAA peak area ratio on ^1^H MRS, (b) will be able to identify infants with severe HIE, and (c) will be able to differentiate the infants with severe HIE from the group with mild to moderate HIE based on both early biomarker and neurodevelopmental assessment at one year of age.

We aimed to investigate the effects of disturbances in brain metabolism following HIE on outcome, using a metabolic reactivity index derived from wavelet analysis between oxCCO and MABP in a cohort of infants undergoing hypothermia (HT) following HIE.

## Material and methods

This prospective observational study (Baby Brain Study) was approved by the Research Ethics Committee (REC) of University College London Hospital and London Bloomsbury REC (reference: 13/LO/0106) in accordance with the declaration of Helsinki. Written informed consent was obtained from parents before each study.

### Patients and clinical care

Stable term infants admitted to the neonatal unit in University College London Hospital for HT following HIE were eligible for the study. Unstable and sick infants or infants with congenital abnormalities were excluded from the study. Clinical decisions regarding the care of the infant were taken by the intensive care team in line with the local and national guidelines. Whole body HT with intracorporeal temperature monitoring was instituted in infants with evidence of moderate to severe HIE as early as possible after birth as per the National Institute for Health and Clinical Excellence (NICE) guidance.^47^ This diagnosis was confirmed by clinical examination, umbilical cord gas acidosis and abnormal electrical activity on electroencephalogram (EEG) or amplitude-integrated electroencephalogram (aEEG). A servo-controlled cooling machine (Tecotherm neo, Inspiration healthcare, UK) was used to maintain the temperature at 33.5℃ for 72 h before gradually increasing the temperature to 37℃ over 14 h.

### Monitoring and data collection

**Figure 1.**
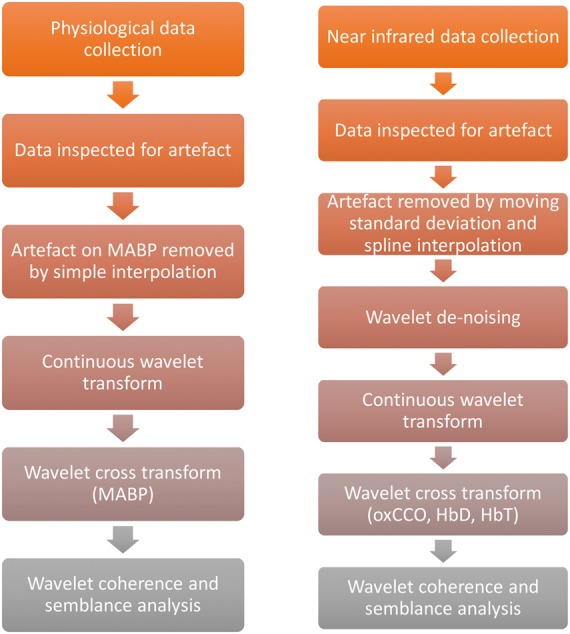
Scheme of data processing and wavelet analysis. Both systemic and NIRS data were checked for artefact after data collection. Artefacts were removed before further processing to reduce the high frequency noise maintaining the trend information. Continuous wavelet transform was performed on both MABP and NIRS data. The wavelet cross transform was then used between these wavelets transform to calculate the measures of power (wavelet coherence) and the instantaneous phase difference (wavelet semblance).

Physiological and broadband NIRS data were collected over 2–4 h, from which a 60-min period from each infant during periods of clinical stability was selected for this analysis. All infants were sedated (continuous intravenous infusion of morphine sulphate), muscle-relaxed (atracurium intravenous infusion) and ventilated during HT. Invasive blood pressure recording was collected continuously from indwelling umbilical arterial catheter. Physiological data from individual patient monitors (Intellivue monitors, Philips Healthcare, UK) were captured using ixTrend software (ixcellence, Germany), down-sampled and synchronised with broadband NIRS timeframe using a MATLAB (MathWorks, Natick, MA)-based software using spline interpolation. NIRS data were collected using a customised software developed in LabView (National Instruments, TX, USA). Figure 1 represents a scheme of data processing and wavelet analysis. NIRS data from both left and right sides revealed similar changes and data from the left side were used for further analysis as thalamic Lac/NAA from MRS were obtained using a single voxel positioned on left thalamus.

### Broadband near infrared spectroscopy

CCO contains four active metal redox centres; one of them, the CuA is a dominant near-infrared (NIR) chromophore and the primary contributor for the NIR spectral signature. Detection of CCO using NIRS is more difficult than other chromophores as its in-vivo concentration is less than 10% of that of haemoglobin and has a broad spectral signature. Broadband NIRS can accurately resolve the spectral changes due to oxCCO without crosstalk from the haemoglobin chromophores. We have recently developed a new broadband NIRS system,^19,22–24^ which is capable of monitoring Δ[oxCCO] as well as Δ[HbO_2_] and Δ[HHb] in the neonatal brain with improved signal quality measured over 136 wavelengths. The system consists of an optical fibre illuminator (ORIEL 77501, Newport, UK) with a stable white light source. The source is coupled to two optical fibre bundles which illuminate the tissue. Four detector bundles collect the attenuated light emerging from the tissue at increasing distances from each source (1.5, 2, 2.5, 3 cm). The optical fibres are held to the tissue in a custom 3D printed holder. At the detection end, a lens-based spectrometer (LS785, Princeton Instruments, USA) and a front-illuminated CCD camera (PIXIS 512f, Princeton Instruments, USA) resolve the intensity spectrum across 770–906 nm for the eight detectors simultaneously. The longest source-detector distance of 3 cm was chosen to ensure an optimal depth penetration and the differential path length (DPF) of 4.99 was used^48^ to calculate the concentration changes of different chromophores using UCLn algorithm.^19^

### Data processing

MABP and NIRS data were visually inspected for any artefacts. Sudden changes in NIRS variables greater than 15% from baseline and not consistent over all the signals were identified as artefacts. Brief transient artefacts in MABP were removed by simple interpolation. Artefacts in NIRS data were removed by using moving standard deviation and spline interpolation in MATLAB.^49^ After artefact removal, NIRS data were processed with an automatic wavelet de-noising function in MATLAB to reduce the high frequency noise but maintain the trend information.

### Slow wave analysis

Keeping in mind the non-stationary aspect of CA, SWs in MABP and NIRS signals were analysed using wavelet-based techniques to achieve high time-frequency resolution. The continuous wavelet transform (CWT) with the complex Morlet wavelet has been shown^50–54^ to be a powerful mathematical tool for time-frequency analysis for both stationary and non-stationary time series. Our group have used this technique in multiple studies^54–55^ including a recent study to illustrate and characterise changes in cerebrovascular reactivity following adult brain injury.^46^ Tian et al.^17^ has also suggested a potential clinical use of this technique to assess the dynamic CA following HIE.

We have used the same MATLAB-based tools described by Highton et al.^46^ to determine wavelet coherence and semblance. Wavelet coherence based on CWT was calculated as a measure of similarity in spectral power and dynamic relationship between spontaneous oscillations in MABP and NIRS variables (oxCCO and HbD). Wavelet coherence varies from 0 to +1 depending on the strength of relationship between the variables. Wavelet semblance was calculated as a measure of instantaneous phase difference and creates an index from + 1 (when the signals vary with close alignment) to −1 (when the signals are completely in antiphase). Wavelet semblance bears a similarity to previously described time-domain indices (PRx, Mx)^28,57^ and gives us the opportunity to assess the cerebrovascular and cerebral metabolic reactivity in a similar fashion.

The haemodynamic (semblance of MABP and HbD or HbT) and metabolic (semblance of MABP with oxCCO) reactivity indices were calculated across a 60-min study period for each infant and the mean values were used for comparison. Indices and variables were documented using median, range or with mean ± standard deviation as appropriate. Datasets were checked for normality using D’Agostino-Pearson omnibus normality test before further statistical analysis in Graphpad Prism 6 (GraphPad, USA). Welch’s correction was performed while comparing between groups when standard deviation was different. Statistical significance was considered as *p* < 0.05.

### Magnetic resonance imaging and spectroscopy

MRI of brain is the imaging modality of choice following HIE and together with MRS, clinically used to assess the injury severity and for prognostication.^11,58^ Thalamic Lac/NAA peak area ratio obtained from ^1^H MRS is a robust quantitative measurement within the neonatal period for prediction of neurodevelopmental outcome following HIE.^10^ Lac/NAA < 0.3 indicated good motor outcome following HIE in this systematic review and meta-analysis. We have used the NICHD neonatal MRI brain injury scoring system described by Shankaran et al.^11^ for prediction of neurodevelopmental outcome at six to seven years of age following HIE. Specific patterns of MRI brain injury 2B (basal ganglia thalamic (BGT), anterior or posterior limb of internal capsule (ALIC or PLIC), or watershed (WS) infarction and cerebral lesions) and three (cerebral hemispheric devastation) in this study were highly predictive of death or IQ < 70 at six to seven years of age.

MRI and ^1^H MRS were performed between day 5 and 7 using a 3 T Philips MRI scanner (Philips Healthcare, UK). T1-weighted imaging was acquired using an inversion-recovery prepared spoiled gradient echo (inversion time = 1465 ms; TR = 17 ms; TE = 4.6 ms; excitation flip angle = 13°). T2-weighted imaging was acquired using 2D fast spin echo (axial and coronal sections; TR = 10721 ms, TE = 130 ms), Diffusion tensor imaging was acquired with 32 directions of diffusion weighting with b-values of 0 and 750. Apparent diffusion coefficient (ADC) and fractional anisotropy (FA) maps were reconstructed inline on the scanner. For MRS, a single PRESS voxel of 1.5 × 1.5 × 1.5 cm was positioned to encompass as much of left thalamus as possible while avoiding overlap with CSF (TR = 2288 ms, TE = 288 ms, 2048 datapoints with spectral bandwidth of 4000 Hz; water suppression was performed using chemical shift selective suppression pulses; automated shimming was performed by the scanner before each acquisition). A dynamic series of 16 subspectra were acquired, each with eight averages. These subspectra were subsequently and individually frequency and phase corrected before summation to yield the final full spectrum.^59^ This methodology allows for any subspectra corrupted by patient motion to be removed from the final summation. NAA, choline, creatine and lactate peaks were identified at 2.02, 3.02, 3.24 and 1.33 ppm, respectively. Spectra were fitted using AMARES^60^ as implemented in the jMRUI magnetic resonance software package.^61^

A paediatric neuroradiologist (RG) scored all MRI images.

### Neurodevelopmental follow up

All infants born in our hospital had regular neurodevelopmental follow-up and were assessed with Bayley Scales of Infant Development-III. Infants born in other hospitals and were transferred to us for management of HIE were also offered the first assessment in our hospital and had the opportunity to choose further assessments in either our hospital or in their local units. A score of <85 was considered adverse outcome (mean 100, SD ± 15). In this study, infants with adverse neurodevelopmental outcome and death were compared with the group who survived with a good outcome at one year of age.

### Blood pressure variability

MABP variability in the SW spectrum (0.003–0.05 Hz) and the relationship with outcome biomarkers were reviewed in the study population. It was important to identify whether MABP variability was directly related to outcome in this cohort and might have influenced the relationship of metabolic reactivity index from wavelet analysis with outcome. This was performed in the frequency domain using established spectral analysis techniques.^62,63^ Power spectral density (PSD) analysis was performed using Welch’s method to determine the power in the SW range and a power index calculated based on SW power/Total power using MATLAB.

## Results

Twenty-three term newborn infants with moderate to severe HIE participated in the study while undergoing HT. Sixty-minute datasets collected during stable periods at a mean age of 48 h were analysed during which transcutaneous CO_2_ (tcCO_2_) remained stable. All infants had MRI and MRS of brain between day 5 and 7 of life. Four infants died either in the neonatal period or within the first year of life. Patient characteristics are presented in Table 1. No significant changes in ventilatory requirements or systemic observations (heart rate, peripheral arterial oxygen saturation (SpO_2_) and MABP) were noted during the study periods.

**Table 1.** Patient characteristics.

Gestational age (weeks+days)	39 (34+2–41+6)
Age at study in hours	48±12
Sex (Male: Female)	1.5:1
Birth weight (g)	3137±426
Arterial cord pH	6.94±0.2
Base deficit	16.05±6.24
Apgar score at 1 min	2±2
Apgar score at 5 min	4±3
Apgar score at 10 min	5±3
Age at study in hours	48±12
Age at MRI (days)	5–7

Note: Gestational age is presented as mean (range) while other parameters are presented as mean± s.d.

MRI: magnetic resonance imaging.

Wavelet coherence and semblance between oxCCO and MABP were calculated as 0.37 ± 0.08 and 0.06 ± 0.13, respectively. HbD-MABP wavelet coherence and semblance were 0.42 ± 0.09 and 0.12 ± 0.17, while HbT-MABP coherence and semblance were 0.38 ± 0.06 and 0.07 ± 0.14. Thalamic Lac/NAA on MRS varied between 0.11 and 2.64.

**Figure 2.**
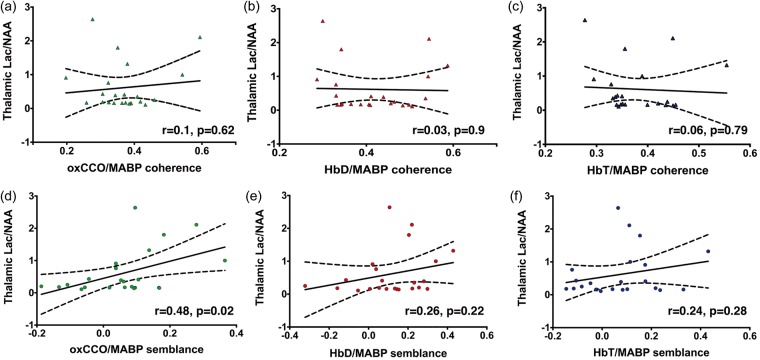
Linear regression analysis between thalamic Lac/NAA and wavelet indices. Note that coherence indices did not reveal any clear correlation with thalamic Lac/NAA (a to c). oxCCO-MABP semblance correlated well with thalamic Lac/NAA (Pearson correlation 0.48, *r*^2^ 0.23, *p* = 0.02) (d). Correlation of thalamic Lac/NAA with HbD-MABP semblance and HbT-MABP semblance were non-significant (*r* = 0.26, *r*^2^ 0.07, *p* = 0.22 and *r* = 0.24, *r*^2^ 0.06, *p* = 0.28) (e, f).

A significant correlation was noted between oxCCO-MABP semblance and thalamic Lac/NAA (*r* = 0.48, *r*^2^ 0.23, *p* = 0.02) (Figures 1(b) and 2(d)). oxCCO-MABP coherence revealed poor correlation with thalamic Lac/NAA (*r* = 0.1, *r*^2^ 0.01, *p* = 0.62) (Figure 2(a)). The relationships of thalamic Lac/NAA with HbD-MABP semblance and HbT-MABP semblance were non-significant (*r* = 0.26, *r*^2^ 0.07, *p* = 0.22 and *r* = 0.24, *r*^2^ 0.06, *p* = 0.28) (Figure 2(e) and (f)). Wavelet coherence of HbD-MABP and HbT-MABP did not correlate with thalamic Lac/NAA (*r* = 0.03, *r*^2^ 0.0009, *p* = 0.9 and *r* = 0.06, *r*^2^ 0.004, *P* = 0.8) (Figure 2(b) and (c)).

**Figure 3.**
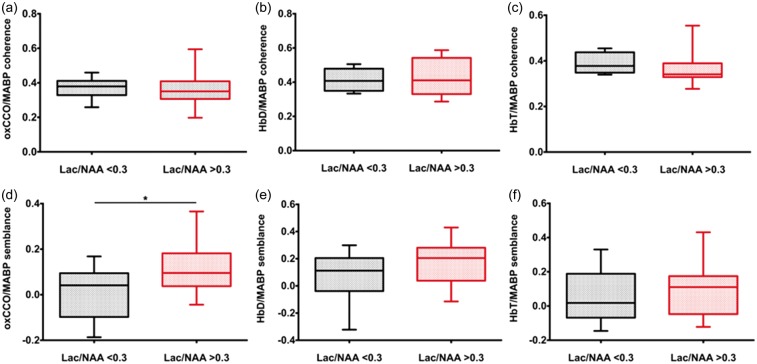
Box and whiskers plots for comparison of mild to moderate and severe groups of infants based on the MRS biomarker (Lac/NAA < 0.3 and Lac/NAA > 0.30) for the wavelet indices. OxCCO-MABP semblance was significantly different between two groups (two tailed *p* 0.04) (d). HbD-MABP and HbT-MABP semblance difference between the groups were non-significant (two tailed *p* = 0.18 and 0.51, respectively) (e, f). Wavelet coherence difference between the groups were also non-significant for oxCCO-MABP (a), HbD-MABP (b) and HbT-MABP coherence (c) (two tailed *p* = 0.96, 0.88 and 0.35, respectively).

Metabolic reactivity as expressed by oxCCO-MABP semblance, was significantly different between mild to moderate and severe groups of infants (based on Lac/NAA < 0.3 and Lac/NAA ≥ 0.3) (two tailed *p* 0.04 (Figure 3(d)). HbD-MABP and HbT-MABP semblance difference between groups were non-significant (two tailed *p* = 0.18 and 0.51, respectively) (Figure 3(e) and (f)). Wavelet coherence difference between the groups was also non-significant for oxCCO-MABP, HbD-MABP and HbT-MABP coherence (two tailed *p* = 0.96, 0.88 and 0.35, respectively) (Figure 3(a) to (c)).

**Figure 4.**
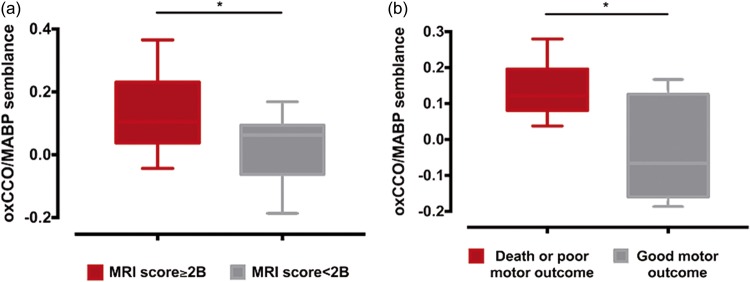
(a) Difference in oxCCO-MABP semblance in two groups of infants with MRI score < 2B (grey) and ≥ 2B (red) (Shankaran et al. 2016) indicating a significant difference (two tailed *p* = 0.04) between the groups (mean and SEM presented). (b) Relationship between the oxCCO-MABP semblance and neurodevelopmental outcome at 12 months of age following HIE. OxCCO-MABP semblance was presented for two groups of infants – death or motor disability at 12 months with Bayley III motor composite score < 85 (red) and normal motor outcome with Bayley III motor composite score > 85 (grey). Significant difference (two tailed *p* = 0.04) noted in oxCCO-MABP semblance between two groups of infants.

OxCCO-MABP semblance compared between two groups based on MRI score (MRI score < 2B and ≥ 2B) demonstrated a significant difference (two tailed *p* = 0.04, effect size (Cohen's d) 0.94) between the groups (Figure 4(a)). Nine infants in this cohort had an MRI score of 2B or more.

Neurodevelopmental outcome data were available up to 12 months of age for 11 infants. Four infants died within the first year. Mean oxCCO-MABP semblance was significant different between two groups (death or Bayley III motor composite score < 85 and Bayley III motor composite score ≥ 85) (two tailed *p* = 0.04, effect size (Cohen's d) 1.52) (Figure 4(b)).

**Figure 5.**
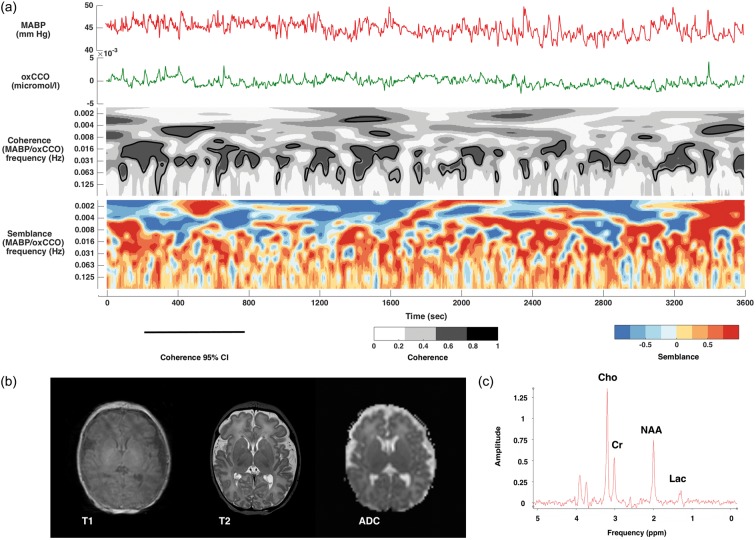
Individual example of wavelet coherence and semblance calculation in an infant admitted with moderate encephalopathy. Mean coherence and semblance for the study were 0.46 and −0.11 respectively ((a). The infant was born in poor condition following fetal bradycardia at 37+6 weeks by emergency caesarean section and was resuscitated at birth. Arterial cord revealed pH 6.99, pCO_2_ 12.81 and BE −10.2 with Apgar score 1 ant 1 min and 5 at 5 min. Infant completed 72 h of HT. There was generalised low signal intensity was noted on the T1-weighted images and high signal intensity on the T2-weighted images on MRI on day 5, but no overt acquired pathology was noted (b). ^1^H MRS-derived Lac/NAA peak area ratio was measured 0.25 with normal choline (Ch), creatinine (Cr), N-acetyl aspartate (NAA) peak and a small lactate (Lac) peak (c).

**Figure 6.**
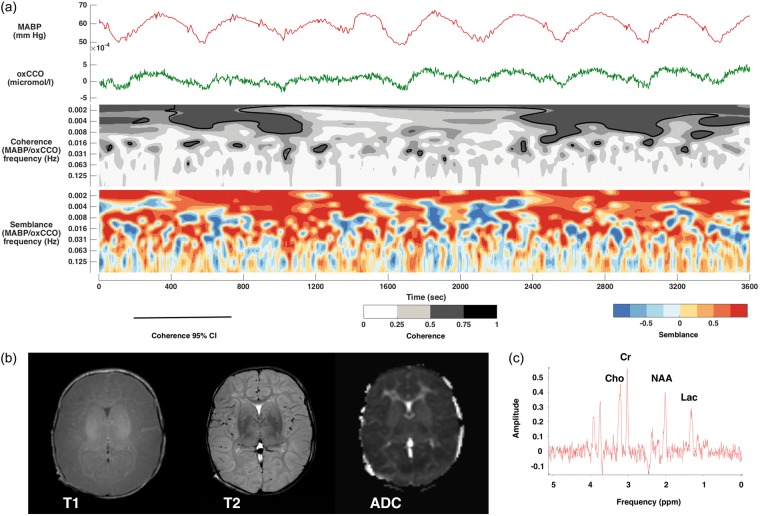
Example of passive oxCCO-MABP reactivity in an infant admitted with severe HIE. (a) Demonstrates calculation of wavelet coherence and semblance between oxCCO and MABP – 0.54 and 0.37, respectively. Note the difference in semblance colour map in contrast to the previous example with intact metabolic reactivity (Figure 6). This infant was born at 41+2 weeks by emergency caesarean section following fetal bradycardia and evidence of thick meconium. Baby was born with no respiratory effort with a heart rate of ∼60/min and needed resuscitation. Arterial cord gas revealed pH 7.09. PCO_2_ 8.30. BE −12.30 and Apgar score was 2 at 1 min, 3 at 5 min. Baby received HT. EEG throughout this period remained significantly suppressed). MRI of brain on day 5 revealed global cerebral swelling and edema with restricted diffusion in keeping with global infarction due to severe HIE (b). ^1^H MRS revealed a split choline peak, a smaller NAA peak and a raised Lac peak. Lac/NAA ratio was 1, also indicating a severe degree of deep grey matter injury (c). Metabolite concentrations are reduced in the thalami of neonates with severe HIE.^88^ compared to normal/mild outcomes. As a result, spectra acquired from the brains of neonates with severe HIE have a lower SNR. This effect can be seen by comparison of Figures 5 and 6.

A strength of wavelet analysis is the possibility to investigate the relationships between MABP and NIRS indices in detail for individual studies. Figures 5 and 6 demonstrate examples of preserved and disturbed metabolic reactivity in two infants with good and adverse outcomes respectively following HIE. MRI images and the MRS spectra for calculation of Lac/NAA are also presented.

The power spectral density analysis of MABP waveform was performed in all infants. Average power in the SW frequency range (0.003–0.5 Hz) with SW/total power index was calculated. No significant differences were noted (two-tailed *p* value 0.26 and 0.93 respectively, Mann–Whitney test) between groups of infants with normal or poor outcome (based on Lac/NAA < 0.3 and Lac/NAA ≥ 0.3, respectively).

## Discussion

This study demonstrates that the metabolic reactivity index, defined as the semblance of oxCCO and MABP at 48 h of age calculated using wavelet transformation, predicted outcome following HIE. This relationship was demonstrated both for short-term outcome biomarker based on MRS-derived Lac/NAA between days 5 and 7 and neurodevelopmental outcome measured at one year. Infants with preserved cerebral metabolic reactivity (low wavelet semblance ranging from 0 to –1) had better outcome compared to infants with disturbed metabolic reactivity (high wavelet semblance 0 to +1). Variability of MABP itself did not influence the relationship of oxCCO-MABP semblance with outcome. OxCCO-MABP wavelet coherence as well as other wavelet indices between MABP and haemoglobin-based indices (HbD and HbT) did not correlate with outcome biomarkers and did not differ among groups of newborn infants with death or poor outcome and normal outcome. Metabolic reactivity index (oxCCO-MABP semblance) appears to be a promising cot side indicator of outcome following HIE.

OxCCO-MABP semblance is likely to reflect the metabolic response to blood flow changes and substrate delivery to mitochondria and as a result becomes a marker of metabolic reactivity. Our findings suggest that oxCCO-MABP semblance can identify those neonates where impaired CA is compromising flow-metabolism coupling. Therefore, it is related to outcome more closely than HbD and HbT indices from wavelet analysis. Wavelet coherence indicates where signal powers vary together between the variables and reflects change in power and synchronisation of phase. In contrast, wavelet semblance gives a measure of phase difference, making it a more effective index for assessment of cerebral reactivity and autoregulation. The phase dynamics derived from wavelet analysis between MABP and cerebral blood flow velocity (CBFV) reflected most of the linear and non-stationary characteristics of CA in a study by Latka et al.^53^ This corroborates with our findings of oxCCO-MABP wavelet semblance being a superior index of reactivity compared to wavelet coherence.

CA in the newborn brain attempts to maintain a constant CBF over a range of perfusion pressure and a key protective mechanism.^64^ Pressure passivity of the cerebral circulation has been documented in sick term^16^ and preterm^29^ infants and has been related to outcome in both newborns^14^ and adults.^65^ Recent studies in newborn term infants following perinatal brain injury have suggested the importance of early identification of loss of autoregulation indicating the need for continuous monitoring with appropriate indices of cerebrovascular reactivity.^13–17^

Metabolic regulation of blood flow is well established. CBF and cerebral metabolism are tightly controlled in the healthy brain,^66^ but this relationship is likely to be disturbed following HIE. Cerebral nitric oxide (NO) maintains cerebrovascular tone by modulating CBF.^67^ Under normal conditions, intra-mitochondrial Ca++ activates mitochondrial phosphates, which in turn activates cytochrome c and CCO. Following HIE, increased Ca++ influx inside cell activates neuronal nitric oxide synthase (nNOS) stimulating the production of NO from L-arginine and oxygen.^68^ NO disrupts the mitochondrial respiratory chain by impairing the function of CCO (complex 4) and complex 1 and induce apoptosis.^69–73^

NO-mediated injury pathways may explain our findings of passive (zero to +1) cerebral metabolic reactivity (oxCCO-MABP semblance) in infants with severe perinatal brain injury and poor outcomes. It is likely that increase in cerebral NO production following hypoxic ischaemic injury is responsible for the haemodynamic changes as well as the secondary energy failure by inhibition of mitochondrial respiration at the level of CCO.

Two periods of vasoparasis have been identified following HIE. The initial phase happens soon after the hypoxic ischaemic insult and the second phase 12–24 h later, which continues for hours or days depending on the injury severity.^27,29,74,75^ This second phase of increased cerebral perfusion is related to the secondary energy failure,^29,76^ during which an increase in extracellular concentration of citrulline^77^ has been documented in late gestation fetal sheep. Citrulline is produced during the production of NO from L-arginine by NOS, suggesting an increased production of NO during this period. NO induces cerebral vasodilation as well as neuronal death through free radical injury. Relationship of oxCCO-MABP semblance at 48 h with outcome most likely reflects the effect of established mitochondrial injury and vasoparasis at this point. It is interesting to note that inhibiting NOS following cerebral ischaemia in fetal sheep increased cerebral injury,^78^ most likely by limiting the substrate delivery to ETC within the already compromised mitochondria.^79,80^

Monitoring cerebrovascular reactivity and assessing its relation to outcome has been an important focus following newborn brain injury. A series of different methodologies have been used to directly measure CBF – Doppler ultrasound,^81^ Xenon-133 clearance,^82^ (positron emission tomography (PET),^83^ single photon emission computed topography (SPECT)^84^ and perfusion-weighted MRI.^85^ But these techniques are not suitable for continuous monitoring at cot side in the neonatal intensive care. NIRS has emerged as an alternative tool for monitoring cerebrovascular reactivity at the cot side.^13–17^ Identification of an optimal blood pressure was examined in a group of infants with HIE to find the optimal vasoreactivity.^16^ Infants with significant injury following HIE spent a greater proportion of time below optimal mean arterial blood pressure (MAP_OPT_) and had a greater deviation from MAP_OPT_ compared to infants with normal outcome. Time spent below MAP_OPT_ was also associated with neurodevelopmental impairment at two years. Massaro et al.^15^ demonstrated a similar relationship using pressure passivity index (PPI), an indicator of duration of pressure passive circulation.^15^ These methodologies were based either in time or frequency domain. Several studies have looked at the cerebrovascular reactivity but the metabolic regulation or metabolic reactivity indices has long been overlooked.

One of the major challenges for signal-processing for analysis of CA is the dynamic and non-stationary nature of cerebral slow-waves. Our group and recently Tian et al.^17^ have demonstrated that using wavelet-based tools, it is possible to characterise CA better. Tian et al. described the time-scale-dependant nature of CA with demonstration of in-phase and antiphase coherence and their relationship to outcome following HIE. Wavelet semblance in this study examined the phase differences between MABP and NIRS indices. It is the cosine of the instantaneous phase difference and varies toward +1 when signals are closely aligned in phase and toward −1 when they are in antiphase. Wavelet semblance^46^ being in a scale of −1 to +1 can be used more intuitively, like time domain-based reactivity indices.^45,86^ The individual colour maps for wavelet coherence and semblance also help to understand the cerebral physiological changes over time in individual cases (Figures 5(a) and 6(a)). Recently real-time wavelet analysis has been used to study neurovascular coupling (NVC) in neonatal encephalopathy. NVC coherence between NIRS measured cerebral tissue oxygenation and EEG was lower in cooled encephalopathic infants compared to the non-encephalopathic group. The coherence was also significantly lower in the poor outcome group compared to those with a normal outcome.^87^ Although the broadband NIRS-derived marker of mitochondrial metabolism (oxCCO) and EEG are two different physiological measurements and a direct comparison between them is not possible based on our study, it is interesting to note that both wavelet approaches were able to differentiate between the poor and normal outcome groups after HIE.

## Limitations

A significant proportion of infants admitted to our unit for management for HIE were transferred ex-utero from other local units. We could not obtain consent for study on day 1 for these infants as parents were not available. Findings of this study represent the effect of hypoxic ischaemic injury on cerebral metabolism at 48 h of life during HT. To interrogate the role of this metabolic reactivity index for understanding of the pathophysiological changes following HIE, measurements need to be taken early after hypoxia ischaemia and at regular intervals during HT and rewarming. Cerebrovascular reactivity can be affected by changes in pCO_2_, arterial oxygen saturation, CBF, CBV and cerebral oxygen consumption. We have tried to keep these variables constant by choosing periods of clinical stability. We have measured transcutaneous CO_2_, SpO_2_ and other systemic variables continuously during the studies and selected 60-min epochs when these variables remained mostly stable with minimal changes. We also did not have the neurodevelopmental outcome for all infants and follow up at two years would be optimal, but it was encouraging to note the clear relationship between the metabolic reactivity index at 48 h and neurodevelopmental outcome at one year of age in our cohort.

## Conclusion

Cerebral metabolic reactivity following HIE, as quantified by oxCCO-MABP semblance using wavelet analysis characterised and quantified cerebral metabolic changes in babies with HIE. This reactivity index, oxCCO-MABP semblance was associated with outcome biomarkers used for early prognostication of outcome after HIE as well as the neurodevelopmental outcome measured at one year of age. These findings support the feasibility of wavelet-based assessment of dynamic changes in cerebral metabolism and haemodynamics in newborn infants and the role of oxCCO-MABP semblance as a useful cot side biomarker to differentiate between the infants with good and poor outcome following HIE.
